# The American Text-Book of Obstetrics

**Published:** 1903-06

**Authors:** 


THE AMERICAN TEXT-BOOK OF OBSTETRICS.—In two volumes. Edited by Richard C. Norris, M. D.; Art Editor, Robert L. Dickinson, M. D. Second edition, thoroughly revised and enlarged. Two handsome imperial octavo volumes of about 600 pages each; nearly 600 text-illustrations, and 49 colored and half-tone plates. Per volume: Cloth, $3.50, net; sheep or half Morocco, $4.00, net.

The first 87 pages are devoted to a consideration of the anatomy of the pelvis and generative organs.

The Physiology of Pregnancy is dealt with in a masterly style, and very instructively illustrated, the illustrations including colored plates fully depicting fœtal development and the accompanying maternal changes.

Considerable space is devoted to the "Pathology of Pregnancy," and to a discussion of the best methods of dealing with the pathological conditions. This important subject has, in many books, been dealt with far too lightly.

The last 185 pages of volume I are devoted to the "Physiology and Mechanism of Labor."

Volume II deals with "Dystocia, the Pathology of the Puerperium, the New Born Infant and its Pathology and Physiology, and Obstetric Surgery."

This new edition is amply and well illustrated and contains a record of bacteriologic and chemico-bacteriologic research applicable to the pathology of midwifery.

The wider range of Surgery in treating many of the complications of pregnancy, labor and puerperal period, embraces new problems in obstetrics, some of which have found their place in obstetric practice.

We strongly recommend the American Text-Book of Obstetrics, believing it to be the leading text-book in this branch of medicine.

